# Fast Declining Prediction in Alzheimer’s Disease from Early Clinical Assessment

**DOI:** 10.2174/011570159X332930240925095423

**Published:** 2024-10-28

**Authors:** Lourdes Álvarez-Sánchez, Mar Peretó, Lorena García-Vallés, Ángel Balaguer, Carmen Peña-Bautista, Laura Ferré-González, Miguel Baquero, Consuelo Cháfer Pericás

**Affiliations:** 1 Alzheimer Disease Research Group, Instituto de Investigación Sanitaria La Fe, Valencia, Spain;; 2 Division of Neurology, University and Polytechnic Hospital La Fe, Valencia, Spain;; 3 Faculty of Mathematical Sciences, University of Valencia, Burjassot (Valencia), Spain

**Keywords:** Alzheimer’s disease, neuropsychology, follow-up, early assessment, prognostic, biomarker

## Abstract

**Introduction:**

The heterogenicity in Alzheimer’s Disease (AD) progression hinders individual prognosis. The present work is an observational 2-year longitudinal study in patients with mild cognitive impairment due to AD (n = 52, with positive CSF biomarkers). The aim of this study is to predict which patients are at risk of fast progression. For this, 3 neuropsychological tests based on different domains (clinical dementia, cognition, delayed memory) and the sum of them were used.

**Methods:**

The tests were performed at diagnosis time (T1) and two years after the diagnosis time (T2). Then, the corresponding progression models were developed using each individual test and their sum as a variable response.

**Results:**

As a result, the model based on cognition status to predict fast decline (differences in the Z score (T2-T1) <1.5 were considered fast declining) provided satisfactory performance (AUC 0.74, 83.3% of sensibility and 70.2% of specificity); the models based on clinical dementia and delayed memory to predict fast declining showed low AUC and sensitivity. Nevertheless, the model based on the sum of the 3 tests showed the highest AUC (0.79), low sensitivity (63.6%), and high specificity.

**Conclusion:**

The developed progression models could provide useful information to clinicians and AD patients regarding their fast/normal decline in general or specific domains.

## 1 INTRODUCTION

Alzheimer’s disease (AD) is the most prevalent neurodegenerative disease in the worldwide geriatric population [1, 2]. The cause of the disease is not well understood yet, but the current consensus is a multifactorial etiology in which genetic and acquired factors participate [3]. The biological signs of the disease are the extracellular deposits of non-soluble amyloid-β (Aβ) peptide fibrils and subsequently plaques and the intracellular neurofibrillary tangles of phosphorylated tau (p-Tau) [3-5]. The biological changes and the neurodegenerative process begin years before the first clinical symptoms appear, representing a large clinical phase [6-10]. These biological changes can be detected by using biomarkers (amyloid β42 (Aβ42), tau phosphorylated (p-Tau181), and total tau (t-Tau)) in cerebrospinal fluid (CSF) or positive amyloid-β deposit in brain cortex, measured by PET-TC [11]. Therefore, Aβ pathology biomarkers (Aβ42, Aβ40, ratio Aβ42/Aβ40 [12], amyloid-PET [13, 14] and tau pathology biomarkers (p-Tau and t-Tau [12, 15]), are used for diagnosis of the disease in patients with cognitive impairment.

The most accepted hypothesis for disease progression [16, 17] is based on the Aβ cascade. In this sense, the classification model proposed by the research framework for preclinical AD [18, 19] describes the dynamic process of the disease from biomarkers positivity (A/T/N). Based on the NIA-AA classification, the AD continuum starts with a large stage called preclinical, characterized by positive biomarkers and cognitive impairment onset [20]. Then, the mild cognitive impairment (MCI) stage is characterized by the positivity of the biomarkers plus cognitive impairment that does not fulfill dementia criteria [21]. After that, patients with MCI due to AD progress to dementia stages [22]. Some biomarkers (*e.g*., brain atrophy measured by volumetric magnetic resonance imaging (MRI) [23] and neurofilament light (NfL) levels [24]) could be predictors of future AD progression, but they are non-specific, invasive, and expensive. As an alternative, a complete neuropsychological evaluation could be a useful tool to detect cognitive alterations in the prodromic AD stages [25, 26]. Specifically, neuropsychological batteries with composite scores (normalizing and summing up standardized z scores) [27] have been used to detect changes over the AD spectrum [28, 29]. Among these tests, it is important to highlight the Clinical Dementia Rating (CDR) used to evaluate the general clinical status, mini-mental state examination (MMSE) used to evaluate the cognition impairment, and Repeatable Battery for the Assessment of Neuropsychological Status delayed memory domain (RBANS.DM) used to evaluate the delayed memory impairment.

The rate of AD progression is variable among individuals. Some patients show a classical progression (slow cognitive decline, described as lowering 2-3 MMSE points/year [30]), while other patients show a fast decline or slow decline. In this sense, some studies have tried to define fast decliner patients by using some endpoints such as death or institutionalization [31-33]. Other works have used neurocognitive scales (*e.g*., MMSE) to detect these differences [34] but without biological confirmation of AD [32]. In this sense, there is a lack of longitudinal studies developing methods and biomarkers to predict the AD prognosis in a reliable and personalized way.

The aim of the present study is to evaluate the 2-year performance of MCI-AD patients in some neuropsychological domains (clinical dementia, cognition, delayed memory) or the sum of them in order to identify the patients at risk of suffering from fast decline. For this, some demographical and clinical (neuropsychological tests, biomarkers) variables at diagnosis time were used as predictor variables.

## 2 MATERIALS AND METHODS

### 2.1 Study Design and Participants

An observational 2-year longitudinal study was carried out for prognosis evaluation of AD. Participants were patients from the Cognitive Disorders Unit in the Neurology Service of the Hospital (n = 52). They were diagnosed with mild cognitive impairment due to AD (MCI-AD), by means of some clinical features (CSF biomarkers, neuropsychological evaluation, neuroimaging). At the diagnosis time (T1), the patients who meet clinical criteria of progressive cognitive impairment (reported by patient/relatives/neurologist) were informed about the CSF biomarker testing procedure. Then, a complete neuropsychological evaluation was conducted. It consisted of the following scales: Clinical Dementia Rating (CDR), used to characterize six domains of cognitive and functional performance and a complex battery where multiple cognitive domains were evaluated (RBANS), and a fast and reproducible screening test for cognitive impairment (MMSE). Two years after the diagnosis time (T2) the same neuropsychological evaluation was repeated.

The inclusion criteria for participants were age between 50 and 80 years old, impaired CSF biomarkers (amyloid β42 (Aβ42) or amyloid β42/amyloid-β40 ratio (Aβ42/Aβ40), total tau (t-Tau), phosphorylated tau (p-Tau181)) [11] or positivity in cerebral amyloid PET (performed in patients for whom lumbar puncture could not be done), mildly impaired neuropsychological evaluation, and without impairments in carrying out activities of daily life, according to the National Institute on Aging and Alzheimer's Association (NIA-AA) classification [11].

The exclusion criteria had other neurological disorders that could cause cognitive impairment (brain tumour, traumatic brain injury, epilepsy, brain damage, stroke, multiple sclerosis.) or serious psychiatric pathology (major depression, bipolar disorder, schizophrenia.), severe dementia (independently of the cause) and neurologically unexplorable people (deafness, blindness, illiteracy).

### 2.2 Clinical Assessment

The CSF biomarkers were measured by chemiluminescence (CLIA) immunoassay (Lumipulse^®^Fujirebio, Japan) in the clinical diagnosis service from HULAFE. The cut-off values used for each biomarker were (reference level) <830 pg mL^-1^ for Aβ42, >380 pg mL^-1^ for t-Tau, >60 pg mL^-1^ for p-Tau181, and <0.069 for Aβ42/Aβ40, at least this ratio was altered in MCI-AD participants [35].

The neuropsychological evaluation was carried out by certified neuropsychologists [36]. It included the clinical dementia rating scale (CDR, general functioning scale composed by a global score (CDR.GS), CDR sum of boxes, CDR.M, CDR.O, CDR.JPS, CDR.CA, CDR.HH, CDR.PC) [37, 38]; the Mini-Mental State Examination (MMSE, fast cognition scale for evaluating verbal, memory, and visual-constructional skills) [39, 40]; and the Repeatable Battery of the Assessment of Neuropsychological Status (RBANS, a 12-test set that analysed five cognitive domains: immediate memory (RBANS.IM), visuospatial/constructional (RBANS.V/C), language (RBANS.L), attention (RBANS.A), delayed memory (RBANS.DM)) [41-43], Functional Activities Questionnaire (FAQ) [44], Alzheimer’s Disease Cooperative Study - Activities of Daily Living (ADCS-ADL) [45], Geriatric Depression Scale (GDS) [46]. The cut-off values used for MCI definition were CDR global score (CDR.GS) ≤ 0.5, MMSE score between 24 and 27, and RBANS.DM <85 (at least two altered tests in MCI-AD participants).

Participants’ clinical and biochemical data were obtained at diagnosis time (T1) (ApoE genotype, CSF biomarkers levels, neuropsychological test scores, neuroimaging). Two years later, (T2) neuropsychological test scores were obtained again.

### 2.3 Data Treatment for AD Prognosis Evaluation

Neuropsychological data (CDR sum of boxes, MMSE, RBANS.DM) from T1 and T2 were used to evaluate the AD progression. So, the difference (*diff* = T2-T1) was calculated for each of these variables. In this sense, *diff* CDR sum of boxes, *diff* MMSE, and *diff* RBANS.DM allowed the definition of two AD groups according to their different evaluated domains: the fast decline (class 1, fast decliners) and the normal decline (class 0, normal decliners). For this, different thresholds (Z_th_) in the Z-scores of these *diff* variables were evaluated. Specifically, they were *diff* CDR sum of boxes, *diff* MMSE, *diff* RBANS.DM, the sum of (*diff* CDR sum of boxes + *diff* MMSE + *diff* RBANS.DM. These Z_th_ values were established as a compromise between the smallest possible Z-value distribution for class 1 and a not-very-pronounced imbalance between class 1 and 0. Therefore, the corresponding selected thresholds for each variable were Z_th_ = 1 for *diff* CDR sum of boxes, Z_th_ = -1.5 for *diff* MMSE, and Z_th_ = -1 for *diff* RBANS.DM, Z_th_ = -2 for the sum (*diff* CDR sum of boxes + *diff* MMSE + *diff* RBANS.DM).

### 2.4 Statistical Analysis

A LASSO (least absolute shrinkage and selection operator) penalized logistic regression was developed from the predictor variables at T1 (age, sex, education level, ApoE genotype, CSF p-Tau181, CSF t-Tau, CSF Aβ42, CSF Aβ40, CSF Aβ42/Aβ40, CSF t-Tau/Aβ42, CSF NfL, CDR.GS, CDR sum of boxes, CDR.M, CDR.O, CDR.RSP, CDR.AFC, CDR.ADA, CDR.CP, MMSE, RBANS.IM, RBANS.V/C, RBANS.L, RBANS.A, RBANS.DM, FAQ, ADCS.ADL, GDS), to obtain the prognosis or probability of fast AD decliners by means of different clinical responses. Specifically, the response variables for each developed model were *diff* CDR sum of boxes (model 1), *diff* MMSE (model 2), and *diff* RBANS.DM (model 3), the sum of *diff* CDR sum of boxes + *diff* MMSE + *diff* RBANS.DM (model 4). The LASSO method forces the shrinkage of the parameters to zero, performing predictor variable selection at the model-fitting step. The penalization factor (ʎ) for the LASSO regression in each developed model was determined by cross-validation (k= 3) and maximizing the AUC.

Most of the participants did not show a fast decline in AD, so both classes (0 and 1) were balanced by adjusting their weights (W):

W_0_ = Number of samples / (Samples in class 0 * 2)

W_1_ = Number of samples / (Samples in class 1 * 2)

All these statistical analyses were carried out with the R software (version 4.2.2), R packages lars (version 1.3), and mdatools (version 0.13.1), with IDE R-Studio (version 2022.12.0 Build 353).

The diagnosis indexes (sensitivity, specificity, positive predictive value (PPV), negative predictive value (NPV), and area under the ROC Curve (AUC-ROC)) were obtained for each developed model.

## 3 RESULTS

### 3.1 Demographic and Clinical Description of Participants

Demographic and clinical variables for participants at T1 are summarized in Table **1**. The median age was 71 years, and 65.4% of participants were women. The medians for the neuropsychological test scores at T1 were 23 for MMSE, 3 for CDR sum of boxes, and 52 for RBANS.DM. Therefore, all patients were defined as MCI.

Regarding the follow-up study, the neuropsychological test scores for participants at T2 are summarized in Table **2**. As expected, the CDR sum of boxes increased, while MMSE and RBANS.DM scores decreased over time.

### 3.2 AD Prognosis Evaluation from Neuropsychological Assessment

Four different prognosis models were developed to identify the most relevant demographic and clinical variables to predict the rate of decline of MCI-AD patients according to different neuropsychological domains. In model 1, the increase in the CDR sum of box scores was used as the response variable, and differences in its Z score (T2-T1) ≥ 1 were considered fast declining. In model 2, the decrease in the MMSE score was used as the response variable, and differences in the Z score (T2-T1) < 1.5 were considered fast declining. In model 3, the decrease in the RBANS.DM scores were used as the response variable, and differences in its Z scores (T2-T1) ≤ -1 were considered fast declining. In model 4, the decrease in the sum of the Z scores for each test (to harmonize the direction of the effect, the symbol obtained in the CDR sum of boxes was changed) was used as a response variable, and differences in its Z score (T2-T1) ≤ -2 were considered fast declining.

The most relevant demographic and clinical variables at T1 selected for each developed model are summarized in Table **3**, indicating their corresponding coefficients. As can be seen, sex, RBANS.V/C, and RBANS.L were selected in 3 models, while CDR sum of boxes, MMSE, RBANS.IM, RBANS.DM, FAQ, and some CSF biomarkers (Aβ42, Aβ40, t-Tau/Aβ42) were only selected in one model.

Regarding other relevant variables for prognosis, the ApoE genotype was not selected in any model. However, it is important to evaluate its effect on the fast decline in MCI-AD patients. In this sense, the ApoE status (ε4 homozygous carrier (ε4/ε4), ε4 heterozygous carrier (ε4/ε3), nonε4 carrier) for the fast-decliner patients, which were identified from each developed model, are summarized in Table **4**. As can be seen, 44.4% of fast decliner patients in model 1 were ε4-carriers, and 55.5% were non-ε4 carriers; in model 2, 83.3% of fast decliner patients were ε4-carriers; in model 3, 66.6% were ε4-carriers; and in model 4, 54.54% were ε4-carriers (only 9% of those were ε4-homozygous carriers).

The diagnosis indexes corresponding to the developed models to predict the probability of fast AD decline are summarized in Table **5**. Model 1 showed modest sensitivity and specificity, high NPV (92%) and low PPV (40%); model 2 showed high AUC (0.74), sensitivity (83%) and NPV (97.1%); model 3 showed high specificity (95.7%) and NPV (91.8%), but low AUC, sensitivity and PPV (50%); model 4 showed high AUC (0.79), modesty sensitivity (64%) and high specificity (85.4%) and NPV. In fact, models 2 and 4 showed the highest AUC, model 2 showed the highest sensitivity, and model 3 showed the highest specificity.

## 4 DISCUSSION

In recent years, some research has focused on the advancement in AD prognosis knowledge. Although the use of biomarkers in diagnosis is extended in clinical practice, their use in prognosis prediction remains a challenge. In general, the individual risk of progression could depend on a mix of modulating factors and mechanisms (*e.g*., brain resilience, biological and genetic factors, factors related to immunity, endocytosis, lipid metabolism, and co-pathology [47-51]). Some studies from the literature evaluated the performance of CSF or amyloid PET biomarkers in the preclinical phase and the longitudinal changes in these biomarkers [52, 53], while other studies analyzed the relationship with the onset of cognitive impairment [54, 55].

The present work analysed the capacity to predict which patients are at risk of fast progression using some demographical and clinical (neuropsychological tests, biomarkers) variables as predictors, similar to previous studies [56-58]. In this sense, different neuropsychological tests (CDR sum of boxes, MMSE, RBANS.DM, and the sum of them) evaluating the evolution of some neuropsychological domains were used as response variables in order to predict the 2-year decline in MCI-AD patients. The corresponding developed models showed good accuracy, but the model using the sum of scores (CDR sum of boxes + MMSE + RBANS.DM) as response variable, and some clinical variables (CSF NfL, RBANS.IM., RBANS.V/C, RBANS.L) as predictors, showed the highest AUC (0.79). To our knowledge, this is the first study using the sum of different test scores to predict the general (function + cognition + delayed memory) decline in AD patients, and it showed satisfactory results. Other studies used different tests for prognosis prediction. In fact, Haaksma *et al*. [59] used MMSE and CDR sum of box scores. In this work, they analyzed the differences in scores obtained in three years of follow-up. For this, neuropsychological testing was performed at baseline and a 1, 2, and 3-year follow-up. The authors proposed that the worst score of MMSE and CDR sum of boxes could predict a fast decline in the following years (AUCs of 0.70 and 0.75 for moderate and rapid progression, respectively); so, in this study, the diagnosis of AD was based on clinical criteria [59, 60], without biological biomarkers as predictors. Also, Langbaum *et al*. studied the association of different neurocognitive tests to predict the rate of progression [29], but patients were not diagnosed with biological biomarkers, except in some dementia groups, which were pathologically confirmed by autopsy. Another study from Eldholm *et al*. suggested that test results of CDR sum of boxes, MMSE, and functional measure Instrumental Activities of Daily Living (IADL) at baseline were suitable predictors of progression [61] in a cohort with 16-37 months of follow-up.

Regarding individual tests, the model using RBANS.DM as a variable response showed the lowest AUC (0.54) and sensitivity (33%); therefore, this progression model based on delayed memory impairment would not provide reliable results. The model using MMSE as a variable response showed good AUC (0.74) and satisfactory sensitivity (83.3%) and specificity (70.2%); so, this progression model based on cognition impairment would be a relevant tool to detect a fast decline in cognition. Similarly, previous studies used the MMSE baseline as an outcome for progression prediction. Wilkosz *et al*. [62] analysed some scales and evaluated each year of follow-up in a cohort of 201 patients diagnosed by clinical criteria to identify the differences in clinical decline. They found that baseline MMSE and age were the variables that could better predict the trajectory of the decline. Han *et al*. [63] published a meta-analysis in which it was observed (across 37 studies with 2 years of follow-up) that the baseline MMSE was a promising predictor in AD progression. However, the heterogeneity of the studies and the lack of biomarkers for patients’ diagnoses must be considered. Although MMSE could not be sensitive enough to determine MCI in the earliest stages of the disease [63-65], it is an accessible and reproducible outcome for monitoring the cognitive impairment, even in the dementia stages of AD, while other neurocognitive tests are more sensible but impracticable in moderate stages of dementia (*e.g*., RBANS). The variability of progression in AD is a known factor [66] that affects patients and families. It could be decisive in decision-making when other therapies and clinical trials are developed in studies aimed at detecting fast and slower decliners [67]. In general, those studies found in the literature were based on clinical criteria, without biological biomarkers, being very difficult to differentiate among AD and other neurodegenerative disorders, as well as normal aging. In addition, for the implementation of the developed models, it is mandatory to get neuropsychologists who perform the tests. However, these professionals are not always available in routine clinical practice. In this sense, the potential use of a minimum number of tests at T1 (CDR sum of boxes, RBANS.V/C, RBANS.L, RBANS.A, GDS) to predict the declining rate, and so AD prognosis could simplify the process.

From the biochemical point of view, some CSF biomarkers were selected for the model based on clinical dementia (Aβ42, Aβ40) and for models based on delayed memory and the sum of 3 tests (NfL), indicating that their initial levels could be related to specific and general clinical progression, as it was observed in previous studies [68-73]. Also, the ApoE status was evaluated as a potential factor for fast decline. Actually, some papers pointed out that ApoE ε4-carrier (homozygous or heterozygous) could be a risk factor for “fast decline” in AD [74-77]. Nevertheless, in the present study, ApoE status was not selected as a predictor variable in any model. In fact, only the model based on cognition impairment showed a significantly higher proportion of ApoE ε4-carriers among fast-declining identified patients, but it represented 19% of total ApoE (ε4-carrier) patients.

The main limitation of the present study is the small sample size. Nevertheless, these participants are well characterized by biological CSF biomarkers for AD, identifying patients with amyloidosis accurately. In addition, the same battery of neurocognitive tests was performed at the diagnosis time and two years later. Therefore, the strengths of this study lay in the homogeneous sample of MCI-AD patients, as well as the two-year follow-up data of the same patients, which provide a useful approach to the disease prognosis characterization.

## CONCLUSION

Cognitive and functional characterization of MCI-AD patients who may suffer from fast decline could involve an important advance in prognosis prediction. This work suggests that predictive models based on neuropsychological and clinical variables at the early AD diagnosis time could identify patients with a high risk of fast declining in two years, according to different neuropsychological domains (clinical dementia, cognition, delayed memory), or general impairment. Actually, these progression models could provide useful information to clinicians and potentially benefit patients in access to future treatments. Nevertheless, further research with a higher sample size is required to be carried out to validate these preliminary results.

## AUTHORS’ CONTRIBUTIONS

C.C.P. and M.B. designed the study, L.A.S., L.F.G. and C.P.B. performed the experiments; M.P. and L.G.V. performed the neuropyschological evaluations; A.B. developed the models and analyzed the data; L.A.S and C.C.P wrote the manuscript in consultation with A.B.; all the authors revised the manuscript.

## Figures and Tables

**Table 1:** Demographic and clinical variables for the participants at diagnosis time (T1).

**T1 Variables**	**MCI-AD Participants at T1 (n= 52)**
scope="row">Age (years old) (median (IQR))	71 (67-74)
scope="row">Sex (female, n (%))	34 (65.4%)
Educational level (n, (%)) - No studies - Primary - Secondary - University	1 (1.9%) 31 (59.6%) 11 (21.2%) 9 (17.3%)
ApoE genotype (ε4-carrier (n, %))	ε4/ε4 (5, 9.6%) ε4/ε3 (21, 40.4%) ε3/ε3 (26, 50%)
CDR.GS (scores) (median (IQR))	0.5 (0.5-0.5)
CDR sum of boxes (scores) (median (IQR))	3 (2-3.5)
CDR.M (scores) (median (IQR))	0.75 (0.5-1)
CDR.O (scores) (median (IQR))	0.5 (0.5-0.87)
CDR.JPS (scores) (median (IQR))	0.5 (0.5-1)
CDR.CA (scores) (median (IQR))	0.5 (0-0.5)
CDR.HH (scores) (median (IQR))	0.5 (0-0.5)
CDR.PC (scores) (median (IQR))	0 (0-0)
MMSE (scores) (median (IQR))	23 (20-25.75)
RBANS.IM (scores) (median (IQR))	63 (53-73)
RBANS.V/C (scores) (median (IQR))	75 (62.5-8)
RBANS.L (scores) (median (IQR))	68 (54.75-85)
RBANS.A (scores) (median (IQR))	64 (56-74.25)
RBANS.DM (scores) (median (IQR))	52 (52-86.5)
FAQ (scores) (median (IQR))	5.5 (3-9.75)
ADCS.ADL (scores) (median (IQR))	40 (36-45)
GDS (scores) (median (IQR))	7.5 (5.25-15.50)
CSF Aβ42 (pg mL^-1^) (median (IQR))	598 (468.75-756)
CSF Aβ40 (pg mL^-1^)^a^ (median (IQR))	13911.5 (10504.5-15314.75)
CSF Aβ42/Aβ40 (median (IQR))	0.0485 (0.0393-0.0568)
CSF t-Tau (pg mL^-1^) (median (IQR))	652 (399.25-804.25)
CSF p-Tau181 (pg mL^-1^) (median (IQR))	98 (66.75-141.25)
CSF t-Tau/Aβ42 (median (IQR))	0.92 (0.65-1.43)
CSF NfL (pg mL^-1^)^a^ (median (IQR))	1078.21 (855.86-1324.08)

**Note:** ^a^Aβ40 and NfL were determined in 29 participants. **Abbreviations:** MCI-AD: mild cognitive impairment due to Alzheimer’s disease. IQR: interquartile range. Aβ40: amyloid β40. Aβ42: amyloid β42. p-Tau: phosphorylated Tau. NfL: neurofilament light chain. CDR.GS: CDR global score. CDR.M: CDR memory domain. CDR.O: CDR orientation domain. CDR.JPS: judgment problem-solving domain. CDR.CA: CDR community affairs domain. CDR.HH: CDR home and hobbies domain. CDR.PC: DCR personal care domain. MMSE: mini-mental state examination. RBANS.IM: RBANS immediate memory domain. RBANS.V/C: RBANS visuospatial/constructional domain. RBANS.L: RBANS language. RBANS.A: RBANS attention domain. RBANS.DM: RBANS delayed memory domain. FAQ: functional assessment questionnaire. ADCS.ADL: Alzheimer’s disease cooperative study activities of Daily living. GDS: Geriatric depression scale.

**Table 2:** Neuropsychological variables for participants 2 years after diagnosis (T2).

**T2 Variables**	**MCI-AD Participants at T2 (n= 52)**
CDR sum of boxes (scores) (median (IQR))	5.25 (3-8.75)
MMSE (scores) (median (IQR))	21 (17-24)
RBANS.DM (scores) (median (IQR))	44 (40-60)

**Abbreviations:** MCI-AD: mild cognitive impairment due to Alzheimer’s disease. MMSE: mini-mental state examination. RBANS.DM: Repeatable Battery for the Assessment of Neuropsychological Status delayed memory domain.

**Table 3:** Selected variables for each developed prognosis model.

**T1 Variables**	**Model 1**	**Model 2**	**Model 3**	**Model 4**
Intercept	4.98	3.97	0.31	3.05
Sex	0.28	0.39	-	0.13
CSF Aβ42	-7.5e-04	-	-	-
CSF Aβ40	-1.8e-05	-	-	-
CSF NfL	-	-	8.0e-05	2.3e-05
CSF t-Tau/Aβ42	-	-	0.18	-
CDR sum of boxes	-	0.11	-	-
MMSE	-3.4e-02	-	-	-
RBANS.V/C	-5.2e-02	-7.4e-04	-	-4.8e-03
RBANS.L	-	-3.6e-02	-1.0e-02	-3.8e-02
RBANS.A	-	-1.5e-02	-2.8e-03	-
RBANS.IM	-	-	-	-6.4e-03
RBANS.DM	-	-	1.9e-03	-
FAQ	4.8e-02	-	-	-
GDS	-5.0e-2	-0.17	-	-2.7e-02

**Note:** Model 1: CDR sum of boxes model. Model 2: MMSE model. Model 3: RBANS memory delayed model. Model 4: sum of three tests.

**Table 4:** ApoE status for the fast-decliner patients identified in each model.

**-**	**Model 1 (n=9)**	**Model 2 (n=6)**	**Model 3 (n=6)**	**Model 4 (n=11)**
ε4 /ε4 (n=5)	0	0	0	1
ε4/ ε3 (n=21)	4	5	4	5
ε3/ ε3 (n=26)	5	1	2	5

**Note:** Model 1: CDR sum of boxes model. Model 2: MMSE model. Model 3: RBANS memory delayed model. Model 4: sum of three tests.

**Table 5:** Predictive indexes obtained for each developed prognosis model.

-	**AUC**	**Sensitivity (CI 95%)**	**Specificity (CI 95%)**	**PPV (CI 95%)**	**NPV (CI 95%)**
Model 1	0.68	66.7% (35.4-87.9)	79.1% (64.8-88.6)	40% (19.8-64.3)	91.9% (78.7-97.2)
Model 2	0.74	83.3% (43.6-97)	70.2% (56-81.3)	26.3% (11.8-48.8)	97.1% (85.1-99.5)
Model 3	0.54	33.3% (9.7-70)	95.7% (85.8-98.8)	50% (15-85)	91.8% (80.8-96.8)
Model 4	0.79	63.6% (35.4-84.8)	85.4% (71.6-93.1)	53.8% (29.1-76.8)	89.7% (76.4-95.9)

**Note:** Model 1: CDR sum of boxes model. Model 2: MMSE model. Model 3: RBANS delayed memory model. Model 4: sum of three tests. **Abbreviations:** PPV: Positive predictive value; NPV: Negative predictive value; CI: Confidence interval.

## Data Availability

Not applicable.

## LIST OF ABBREVIATIONS

AD: Alzheimer’s Disease
Aβ: Amyloid-β
CDR: Clinical Dementia Rating
CSF: Cerebrospinal Fluid
GDS: Geriatric Depression Scale
MMSE: Mini-mental State Examination
MRI: Magnetic Resonance Imaging

## STANDARDS OF REPORTING

STROBE guidelines and methodology were followed.
